# Adherence to Personal Protective Equipment Guidelines During the COVID-19 Pandemic Among Health-Care Personnel: A Louisiana Case Study

**DOI:** 10.1017/dmp.2021.176

**Published:** 2021-06-08

**Authors:** Ayushi Aggarwal, Oliver A. Darwish, Mehran Karvar, Chenhao Ma, Mengfan Wu, Valentin Haug, Dennis P. Orgill, Adriana C. Panayi

**Affiliations:** 1 University of Maryland School of Medicine, Baltimore, Maryland, USA; 2 Division of Plastic Surgery, Department of Surgery, Brigham and Women’s Hospital and Harvard Medical School, Boston, Massachusetts, USA; 3 California Northstate University College of Medicine, Elk Grove, California, USA; 4 Department of Plastic and Cosmetic Surgery, Nanfang Hospital, Southern Medical University, Guangzhou, Guangdong, P. R. China; 5 Department of Hand, Plastic and Reconstructive Surgery, Microsurgery, Burn Center, BG Trauma Center Ludwigshafen, University of Heidelberg, Ludwigshafen, Germany

**Keywords:** disease outbreaks, epidemics, occupational exposure

## Abstract

**Objective:**

The aim of this study was to determine the extent that appropriate personal protective equipment (PPE), per Centers for Disease Control and Prevention (CDC) guidance, was used during the coronavirus diseases 2019 (COVID-19) pandemic by health-care personnel (HCP) in Louisiana in 5 clinical settings.

**Methods:**

An online questionnaire was distributed to the LA Nursery registry. Appropriate use of PPE in each of the 5 clinical scenarios was defined by the authors based on CDC guidelines. The scenarios ranged from communal hospital space to carrying out aerosol generating procedures (AGPs). A total of 1760 HCP participated between June and July 2020.

**Results:**

The average adherence in LA was lowest for the scenario of carrying out AGPs at 39.5% compliance and highest for the scenario of patient contact when COVID-19 not suspected at 82.8% compliance. Adherence among parishes varied widely. Commentary to suggest a shortage of PPE supply and the practice of re-using PPE was strong.

**Conclusions:**

Use of appropriate PPE varied by setting. It was higher in scenarios where only face masks (or respirators) were the standard (ie, community hospital or when COVID-19 not suspected) and lower in scenarios where additional PPE (eg, gloves, eye protection, and isolation gown) was required.

During the early stages of the severe acute respiratory syndrome coronavirus 2 (SARS-CoV-2) outbreak in 2019 (coronavirus disease 2019, COVID-19), the sudden increase in demand with limited availability of personal protective equipment (PPE) had placed frontline health-care personnel (HCP) across the nation at higher risks of becoming infected by the virus, hence endangering their health and others who needed their care. Months later, with the second surge of the pandemic, reports of PPE shortages continued to circulate across the country.

Through July, Louisiana (LA) had the highest recorded number of total confirmed cases per capita in the country.^1^ According to the Louisiana Department of Health, since the first presumptive case of COVID-19 in LA on March 9, 2020, the number of cases has spread exponentially across the state.^2^ By April 11, every parish in LA had at least 1 reported case.^3^ The sudden rise in cases could be expected to strain the health-care system and increase the demand for PPE among HCP, especially during a time when the Centers for Disease Control and Prevention (CDC) had updated its guidelines for PPE use in the COVID-19 scenario. This study was conducted to determine the extent to which appropriate PPE was in use per CDC guidelines among nurses in LA.

## Methods

Following the approval from the review board at Brigham and Women’s Hospital (Boston, MA), an online questionnaire was designed using REDCap (Vanderbilt University, TN). Appropriate use of PPE in 5 clinical scenarios was defined by the authors based on CDC guidelines.^4,5^ The clinical scenarios included: communal hospital space, patient contact when COVID-19 is not suspected, patient contact when COVID-19 is suspected, patient contact when COVID-19 is confirmed, carrying out Aerosol generating procedures (AGPs) (Table 1). The survey design was mixed methods. The respondents were asked to type their parish and job title, and select from a list of PPE materials (surgical mask, respirator mask, gloves, etc) they used in each of the 5 clinical scenarios. At the bottom of the survey was the option to leave comments.

**Table 1.** Clinical scenarios and optimal PPE combinations according to CDC guidelines

Setting	Requirements
Communal hospital space	Facemask (or respirator)
Patient contact when COVID-19 is not suspected	Facemask (or respirator)
Patient contact when COVID-19 is suspected	Respirator (or facemask), gloves, eye protection, and isolation gown
Patient contact when COVID-19 is confirmed	Respirator (or facemask), gloves, eye protection, and isolation gown
Carrying out AGPs	Respirator, gloves, eye protection, and isolation gown

The questionnaire was sent to the CEO of the LA Nursing Board, who then distributed the survey to the Nursing Board list serve (29,000 registered RNs and APRNs in 2020).^1^ After the survey was closed, the results were exported into Microsoft Excel 365 (Microsoft, Redmond, WA) and any duplicates were removed. Adherence was determined by comparing the proportion of respondents who selected the proper PPE to our optimum standards. Statistical data processing and visualization were performed using RStudio 1.2.5033 (RStudio, Boston, MA, USA).

## Results

**Figure 1.**
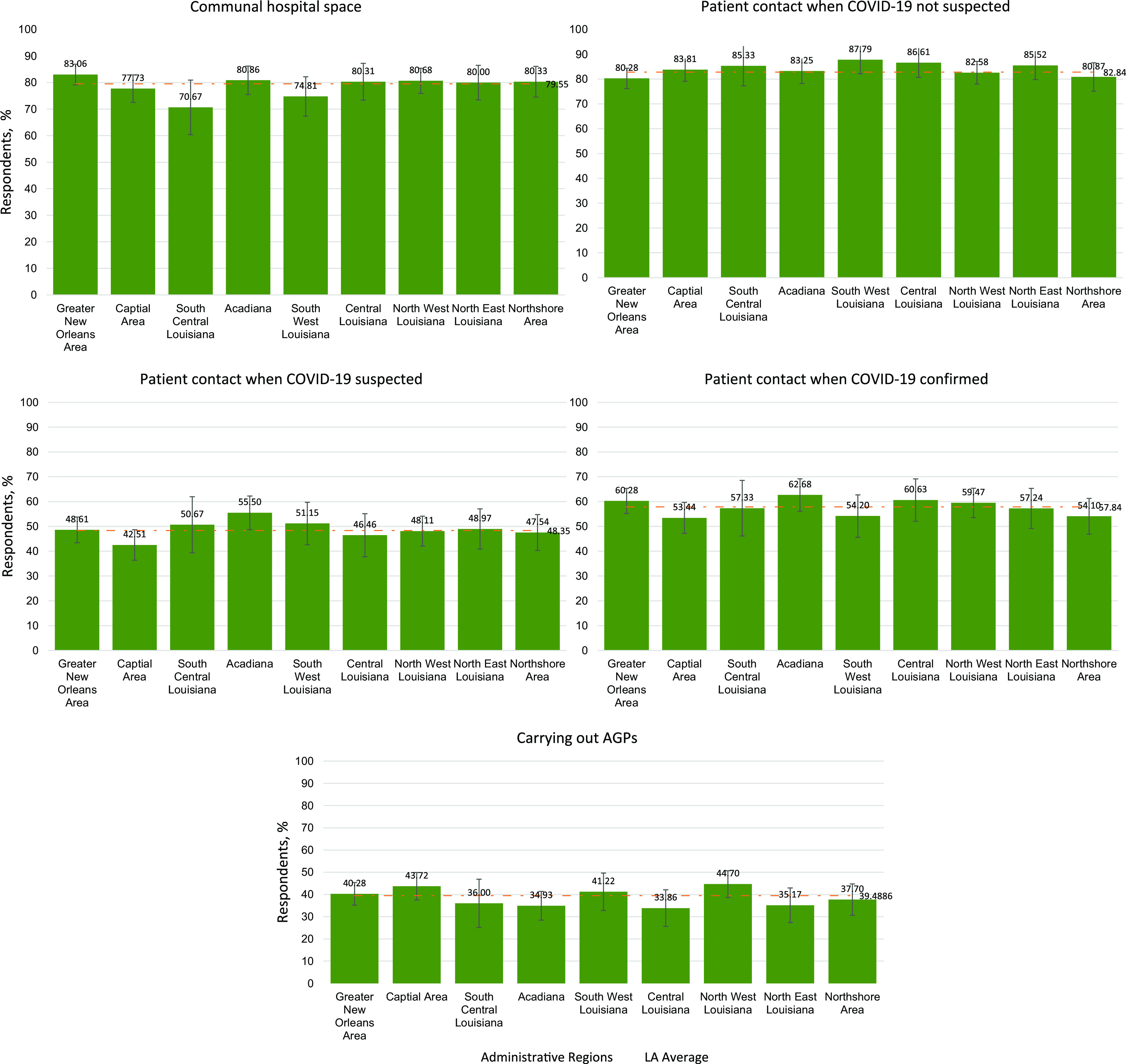
HCP adherence proportions of PPE use under 5 clinical scenarios, by region in Louisiana. Numbers above the bars represent the percentage compliance in each region.

From June 26 to July 17, 2020, a total of 1760 responses from 60 parishes/counties in LA were collected. HCP adherence rates to different scenarios based on the health administrative regions in LA are illustrated in Figure 1. Nine health-care administrative regions were listed in the descriptive analysis. Respondents’ professions included nurse (99.4%), paramedic (0.2%), and medical technician (0.3%).

The average of the levels of adherence in different scenarios were identified as follows: 78.7% for communal hospital spaces, 82.84% for patient contact when COVID-19 not suspected, 48.35% for patient contact when COVID-19 suspected, 57.84% for patient contact when COVID-19 confirmed, and 39.49% for carrying out AGPs. The average adherence in LA was lowest for the carrying out AGPs and highest for patient contact when COVID-19 not suspected scenarios.

In communal hospital spaces, highest adherence was shown by Greater New Orleans Area (83.1%) and lowest by South Central Louisiana (70.7%). South West Louisiana demonstrated highest adherence in the patient contact when COVID-19 not suspected scenario (87.8%), while Greater New Orleans Area demonstrated the least (80.3%). Acadiana showed highest adherence in patient when COVID-19 is suspected (55.5%) and confirmed (62.7%), while Capital Area showed lowest adherence in those scenarios (42.0% for suspected and 53.4% for confirmed). The region with the highest adherence for carrying out AGPs appears to be North West Louisiana (44.7%), while Central Louisiana appears to have the lowest adherence (33.9%).

In addition to the quantitative results, respondents’ commentary concerning the use of PPE in their workplace was compiled and analyzed. There were comments to suggest that “cheap, thin masks are supplied on a 1 mask per week basis” or that “masks are not available and at times, most re-use it.” Most nurses complained about the lack or shortage of PPE provided by their managers and the improper disinfecting of PPE by their institution. Many also felt compelled to reuse PPE and described the difficulties in complying with constantly changing safety protocols in their institution. Remarks such as “the practice of using PPE has come in waves, heightened during the higher influx of COVID cases (March/April and now July) and has loosened during the decrease of numbers (April/May)” and “hospital keeps giving out different guidelines according to supply access” provide more insight into the factors driving PPE use overall.

## Discussion

This study is the first to establish in the literature PPE level of adherence in LA during the COVID-19 pandemic. HCP safety has a direct effect on patient safety, underscoring the value in assessing where gaps in health--care practice exist. Between March and July 2020, LA was the only state to experience 2 spikes of the coronavirus.^6^ The results are reported during a time when the COVID-19 crisis was rapidly evolving within the health administrative regions.

While adherence to the recommended PPE guidelines in different clinical scenarios varied, the results of this survey show that adherence in all relevant health-care scenarios could be improved. Worse adherence is particularly seen in high-risk scenarios, where additional PPE is required and the likelihood of coronavirus exposure is increased: Patient contact when COVID-19 is suspected, patient contact when COVID-19 is confirmed, and carrying out AGPs.

Although our survey did not aim to identify possible reasons for such variability, qualitative comments made by the respondents of the survey highlight some factors driving low adherence rates. These factors include PPE shortages, HCP concerns about PPE re-use, insufficient trainings and/or low level of awareness among HCP, difficulties in using PPE due to pre-existing conditions, the unavailability of good fit PPE (particularly N95 masks), and difficulties to remain up to date with rapidly evolving recommendations following frequent guideline modifications by the CDC or individual institutional administrations.

Differences in adherence patterns among different regions could be attributable to the resources available in the area, the population size, socioeconomic differences, and the institutional commodities. For instance, Acadiana is a region in southern LA with a vast amount of rural territory. At the time of the study, Acadiana consistently had the highest rates of COVID-19 cases. Based on the survey responses, nurses in Acadiana reported the highest adherence in scenarios that require greater protection and pose a higher risk, patient when COVID-19 is suspected and confirmed. It is possible that, in leading the state with COVID-19 cases, Acadiana also had a greater supply of PPE available to combat the progression of the virus.

When considering the limitations of the study, it is important to mention that, with an estimated response rate of 6%, there is the possibility that self-selection occurred. Perhaps nurses who were more cognizant of PPE requirements or more likely to adhere to them were also more likely to respond to the survey. We received comments in the appropriate field that suggested there could also be some selection in the opposite direction from those who were particularly concerned that they did not have the appropriate PPE available to them. Regardless, the survey designed allowed us to reach a mass of nurses in a short amount of time and in a cost-effective way.

### Public Health

Collectively, the results of this survey highlight the critical need to investigate the precise factors that contribute to disparities and low adherence among HCP in LA. Effective educational training programs, an adequate PPE supply, and in-training evaluation are key strategies that can be taken by policy-makers and safety managers of health-care institutions in LA. Further research is required to pinpoint such strategies based on the reasons underlying adherence variability to improve the safety of HCP.

### Policy Implications

As HCP are at the forefront of efforts to contain the coronavirus, the factors underlying variable adherence to CDC protocols in LA need to be further analyzed and addressed.

## References

[1] Johns Hopkins University. COVID-19 dashboard. https://gisanddata.maps.arcgis.com/apps/dashboards/bda7594740fd40299423467b48e9ecf6. Accessed June 16, 2021.

[2] The Guardian. ‘The new gold’: demand for PPE soars again amid shortage as US cases rise. World news. https://www.theguardian.com/world/2020/jun/29/demand-ppe-soars-again-amid-shortage-us-cases-rise. Accessed June 16, 2021.

[3] The Data Center. Monitoring the COVID-19 pandemic in New Orleans and Louisiana. https://www.datacenterresearch.org/covid-19-data-and-information/covid-19-data/. Accessed June 16, 2021.

[4] CDC. Using personal protective equipment (PPE). https://www.cdc.gov/coronavirus/2019-ncov/hcp/using-ppe.html. Accessed June 16, 2021.

[5] European Centre for Disease Prevention and Control. Guidance for wearing and removing personal protective equipment in healthcare settings for the care of patients with suspected or confirmed COVID-19. https://www.ecdc.europa.eu/en/publications-data/guidance-wearing-and-removing-personal-protective-equipment-healthcare-settings. Accessed June 16, 2021.

[6] USA Today. Louisiana: the rare case of a state ravaged twice by COVID-19. https://www.usatoday.com/in-depth/news/2020/08/01/louisiana-second-covid-19-wave-worse-than-first-no-1-per-capita/5558862002/. Accessed June 16, 2021.

